# Maternal Exposure to T-2 Toxin Induces Changes in Antioxidant System and Testosterone Synthesis in the Testes of Mice Offspring

**DOI:** 10.3390/ani10010074

**Published:** 2019-12-31

**Authors:** Jiakun Shen, Aneela Perveen, Niaz Kaka, Zhaojian Li, Pengyuan Dai, Chunmei Li

**Affiliations:** College of Animal Science and Technology, Nanjing Agricultural University, Nanjing 210095, China; shenjiakun2008@126.com (J.S.); annu12_88@yahoo.com (A.P.); aliniazkaka@yahoo.com (N.K.); 2017205017@njau.edu.cn (Z.L.); 2015205015@njau.edu.cn (P.D.)

**Keywords:** maternal exposure, oxidative stress, T-2 toxin, testicular development, testosterone synthesis

## Abstract

**Simple Summary:**

This study investigated the effects of maternal T-2 toxin exposure on the development of testis in the mice offspring. The detrimental effects were assessed by testicular weight, antioxidant capacity, and testosterone synthesis and secretion. Studies have shown that the toxin carried by the mother has bad effects on the testicular development of offspring at puberty, affecting the antioxidant system and testosterone synthesis in the testis, but the maternal exposure of T-2 toxin had no significant impact on the testes of offspring after sexual maturity, suggesting the recovery of reproductive function.

**Abstract:**

T-2 toxin, the most toxic member of trichothecene mycotoxin, is widely distributed in cereals, and has been extensively studied, but few studies focus on the toxicity of maternal exposure to offspring. This study focused on the effects of maternal exposure to T-2 toxin (during gestation and lactation) on the testicular development of mice offspring. Dams were orally administered with T-2 toxin at 0, 0.005, or 0.05 mg/kg body weight from the late stage of gestation to the end of lactation. Testicular samples of the mice offspring were collected on the postnatal day 21, 28, and 56. The results showed significant decreases in body weight and testicular weight on the postnatal day 28. Moreover, significant inhibition of antioxidant system and testosterone synthesis was detected on the postnatal day 28. Furthermore, there were significant decreases in the gene expression levels of *StAR* and *3β-HSD*, which are involved in testosterone synthesis. In general, present results demonstrated that maternal exposure to T-2 toxin during gestation and lactation led to bad effects on the capacity of antioxidant system and inhibited testosterone synthesis in testes during pre-puberty with no significant effects on post-puberty.

## 1. Introduction

T-2 toxin is a trichothecene mycotoxin with high toxicity and produced by various fungus *Fusarium* sp. including *F. soprotrichioides*, *F. poae*, and *F. acuinatum* [1]. T-2 toxin has been widely investigated due to its wide distribution in contaminated cereals such as corn, oats, barley, wheat, rice, and beans. In a global report, the detection rate of T-2 toxin in 27,850 agricultural products from 100 countries was 19%, and the average pollution level was 22 μg/kg [2]. In China, the detection rate of T-2 toxin in feed samples was as high as 79.5%, and the highest content was 735 μg/kg [3]. The association of T-2 toxin with agricultural damage and its toxic effects on animals have been reported in different countries of the world [4,5]. T-2 toxin can cause emesis, diarrhea, and hemorrhage, reducing the weight and inhibiting the immunity of animals [6,7,8]. T-2 toxin can trigger damage in tissue such as cartilage or brain, inducing cell death and apoptosis [9,10,11]. The details of toxicities of T-2 toxin have been well reported in previous reviews [1,12].

Over the past few decades, environmental toxicant exposure has increased throughout the world, leads to infertility, reduced sperm quality, and male reproductive system abnormalities, and has attracted global attention [13]. The continued decline in male fertility was multifactorial, in part result from the exposure to mycotoxins [14]. As widely occurring natural pollution, T-2 toxin was widely distributed in various crops and could infect humans and animals through various pathways [1]. Reproductive dysfunction was the major detrimental effect of T-2 toxin [15]. In recent years, the research of T-2 toxin on the reproductive system has gradually increased and the effects of direct exposure to T-2 toxin on reproduction have been reported in several studies [16,17]. It is of great practical significance to reveal the toxicity mechanism of T-2 toxin on the male reproductive system.

The growth of spermatogenic cells and the secretion of testosterone by Ledig cells are two important events during the testicular development, and both are closely related to testicular health [18]. The antioxidant system is an important regulatory system in testis to maintain spermatogenesis and testosterone synthesis [19]. Spermatogenesis and steroidogenesis are sources of reactive oxygen species (ROS), the powerful antioxidant system in the testes protects cells from oxidative damage [20]. Therefore, the indicators in the antioxidant system can be used to evaluate testicular spermatogenesis and testosterone secretion in testis.

Maternal nutrition is very important for the development of offspring. During pregnancy, the dams transmit nutrients through the umbilical cord or affect the development of fetus through the endocrine system [21]. During lactation, dams feed their offspring through breast milk which plays an important role in the early development of offspring. Therefore, the maternal effects on the offspring persist from gestation to weaning, which indicates a long-term impact has existed in offspring. Feeds contaminated with T-2 toxin, which was metabolized in the mother or milk, have been considered a potential threat to the health of future generations [22]. Weaning and puberty are important periods in the development of the reproductive system of mammals. They may be more sensitive to the effects of environmental toxins, which cannot be ignored. The toxicity and side effects of T-2 toxin have been reported in pregnant mice and their fetuses [23,24]; however, the knowledge of T-2 toxin on the reproductive system of male mice offspring from weaning to sexual maturity was still limited. The object of this study was to investigate the toxicity of maternal exposure to T-2 toxin on testicular development of offspring through the evaluation of antioxidant system, testosterone synthesis, and apoptosis in testis. We look forward to providing useful information for the maternal toxicity of T-2 toxin and giving a suitable method to study the influence of maternal on the reproduction of offspring.

## 2. Materials and Methods

### 2.1. Chemicals

T-2 toxin was purchased from Bailingwei Technology Co Ltd. (Beijing, China). Corn oil was provided by the Aladdin Corporation Ltd. (Shanghai, China) and used to dissolve T-2 toxin.

### 2.2. Experimental Animals and Ethical Approval

Eight-weeks-old healthy Institute of Cancer Research mice (29–30 g male and 25–28 g virgin/female) were purchased from Qinglongshan Laboratory Animal Company (Nanjing, China) and fed in the experimental animal center in Nanjing Agricultural University (Nanjing, China). The mice were housed in cages under hygienic measures and standard conditions (23 ± 2 °C, a relative humidity of 50 ± 10%, 12 h of a light-dark cycle with ad libitum access to feed and water). They were allowed to adapt to the new surroundings for 1 week before the formal experiment. The experimental protocol was approved in accordance with the Guide for the Care and Use of Laboratory Animals prepared by the Institutional Animal Care and Use Committee of Nanjing Agricultural University (permit number SYXK (Su) 2011–0036).

### 2.3. Experimental Design

The environmentally adapted male and female mice were put into in the same cage at a ratio of 1:2 and mated daily from 6:00 p.m. to 7:00 p.m. On the next day morning, all females were examined by vaginal embolization test and vaginal smears to confirm sexual intercourse. The fertilized females were identified and then separated into individual cages, which was identified as the first day of pregnancy. Thirty pregnant mice were randomized into three groups (n = 10/group). After the second week of pregnancy, mice were exposed to T-2 toxin by oral gavage each day with 0.005 (low dose) or 0.05 (high dose) mg/kg of T-2 toxin, respectively, and the control group was treated with the same volume of corn oil, all pregnant mice were treated at 8 a.m. for 28 days. T-2 toxin doses used in this experiment was based on 1% or 0.1% of the LD50 [25,26] to ensure that pregnant mice could survive during the experiment. The offspring were weaned at 21 postnatal days (PND 21), and entered the pre-puberty at PND 28, the post-puberty, at PND 56.

### 2.4. Sampling

At PND 21, PND 28, and PND 56, five male offspring from each group were weighed and then euthanized by cervical dislocation. The testes were excised and weighed. A subset of the testes was fixed in 4% paraformaldehyde solution for histological examination and the remaining testes were stored at −80 °C for antioxidant indicators measurements and real-time polymerase chain reaction (RT-PCR) analysis.

### 2.5. The Measurement of Body Weight and Relative Testicular Weight

After cervical dislocation of mice, their testes were dissected, and their weight was recorded on an electronic balance. The relative testicular weight can be calculated as follow:

Relative testicular weight = (weight of the testis/weight of the mice) × 100%(1)

### 2.6. The Histopathological Examination and Measurement of Seminiferous Tubules Components

**Figure 1:**
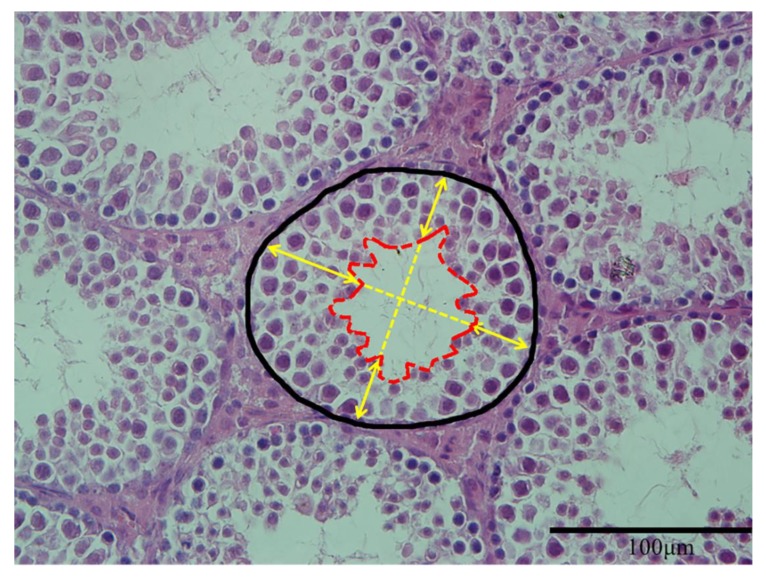
Measurement of seminiferous tubules components. The area in black line defines the cross-sectional area of a seminiferous tubule. The area in red line defines the area of lumen. The average length of the four yellow arrows represented the height of seminiferous epithelium (magnification: ×400).

For histopathological examination, the testes of offspring were fixed in 4% paraformaldehyde solution for 24 h and dehydrated through a graded series of ethanol, cleared in xylene before embedded in paraffin blocks. The blocks were sectioned perpendicular to the longest axis of the testes at a thickness of 5 μm and then kept them dry for 6 h at 42 °C. Sections were stained with hematoxylin and eosin (HE). The stained sections were mounted and examined under virtual light microscopy (Model BX51TF, Olympus Corporation, Tokyo, Japan). Representative indicators of seminiferous tubules components measured in the present study included the cross-sectional area of seminiferous tubules and the height of seminiferous epithelium. Three samples were measured in each group, and five sections were selected from each sample. Both indicators were calculated 10 round tubules (exclude tubules with completely broken epithelium) per section using Image J (NIH, Bethesda, MD, USA). The measurement of seminiferous tubules components was performed by a well-trained researcher using Image J according to the method of previous studies [27,28] with a small modification (Figure 1).

The details were listed as follows: the area from the base to the lumen center was recorded as the cross-sectional area of seminiferous tubules; the distance from the basal membrane to the luminal border was considered to be the height of seminiferous epithelium, and four epithelial heights perpendicular to each other were calculated, and the mean was calculated as the height of seminiferous epithelium of this tubule.

### 2.7. The Measurement of Testosterone in Testes

Testes testosterone level was measured using an enzyme-linked immunosorbent assay (ELISA) (Yifeixue Bio Tech, Nanjing, China) on a multi-function microplate reader (Spark, TECAN, Männedorf, Switzerland) according to the manufacturer directions. In brief, tissue samples were smashed by homogenate in phosphate buffer saline (Solarbio, Beijing, China) using bead mill homogenizer (Bead Ruptor 12, OMNI, Kennesaw, GA, USA) and centrifuged at 3000 rpm for 15 min at 4 °C. The supernatant was collected and measured the concentration of total protein immediately (each sample two replicates) and then diluted in the assay buffer solution provided in the kit and frozen at −20 °C until analysis.

### 2.8. The Measurement of Antioxidant Indicators and DNA Damage in Testes

To determine the capacity of antioxidant system in testis, the testis homogenate was centrifuged to obtain the supernatant (3000 rpm, 15 min and 4 °C), and then the content of malondialdehyde (MDA), the activity of catalase (CAT), superoxide dismutase (SOD) and glutathione peroxidase (GSH-Px) in the testes were measured by assay kits (Nanjing Jiancheng Bioengineering Institute, Jiangsu, China). The DNA damage was detected by 8-hydroxy-2′-deoxyguanosine (8-OHdG) analysis using an ELISA assay (Meimian Co., Ltd., Shanghai, China) on a multi-function microplate reader (Spark, TECAN, Männedorf, Switzerland).

### 2.9. The RT-PCR Analysis of Genes Related to Testosterone Synthesis and Apoptosis

Approximately 50 mg of testes were used for total RNA extraction using a total RNA extraction kit (Vazyme BioTech Co., Ltd., Nanjing, China). The RNA concentration and purity were determined spectrophotometrically at 260 and 280 nm with a Nanodrop^®^ 2000 (Thermo Fisher Scientific, Wilmington, DE, USA). Then, HiScript^®^ II Q RT SuperMix (Vazyme BioTech Co., Ltd., Nanjing, China) was used to reverse transcribe RNA to cDNA. Quantitative real-time PCR was conducted with QuantStudio^®^ 5 Real-Time PCR Instrument (Applied Biosystems, Thermo Fisher Scientific, Wilmington, DE, USA) using the SYBR Green I ^®^ T5 Fast qPCR Mix kit (TSINGKE BioTech Co., Ltd., Beijing, China). Primer sequences for genes related to testosterone synthesis and apoptosis were presented in Table 1. Each RT-PCR was performed with four biological replicates. The relative mRNA expression levels were determined using the 2^−△△CT^ method with β-actin serving as the housekeeping gene [29].

### 2.10. Statistical Analysis

All the data were presented as the mean ± standard error of the mean (SEM) and were analyzed by one-way analysis of variance (ANOVA) followed by Dunnett’s multiple comparison test. The statistical analyses were performed with GraphPad Prism 7.0 software (San Diego, CA, USA). Differences were statistically significant when *p* < 0.05 or *p* < 0.01.

## 3. Results

### 3.1. Effects of Maternal Exposure to T-2 Toxin on the Body Weight and Testicular Weight of Offspring

At all stages, maternal exposure to T-2 toxin induced a decrease in body weight and testicular weight in mice offspring. Among them, the high dose of T-2 toxin significantly reduced body weight and testicular weight in PND 21; both toxin groups significantly reduced body weight and testicular weight in PND 28; while only the high dose of T-2 toxin significantly reduced body weight and testicular weight in PND 56.

After the effect of weight loss was removed, we only found that maternal exposure to the high dose of T-2 toxin significantly reduced relative testicular weight in PND 28, with no significant differences in the remaining stages (Table 2). It suggested that the effects of maternal exposure of T-2 toxin on testicular weight loss were associated with weight loss and it was speculated that mice gradually return to normal reproductive system development (PND 56) and the T-2 toxin may be eliminated by metabolism.

### 3.2. Effects of Maternal Exposure to T-2 Toxin on the Testicular Histology of Offspring

**Figure 2:**
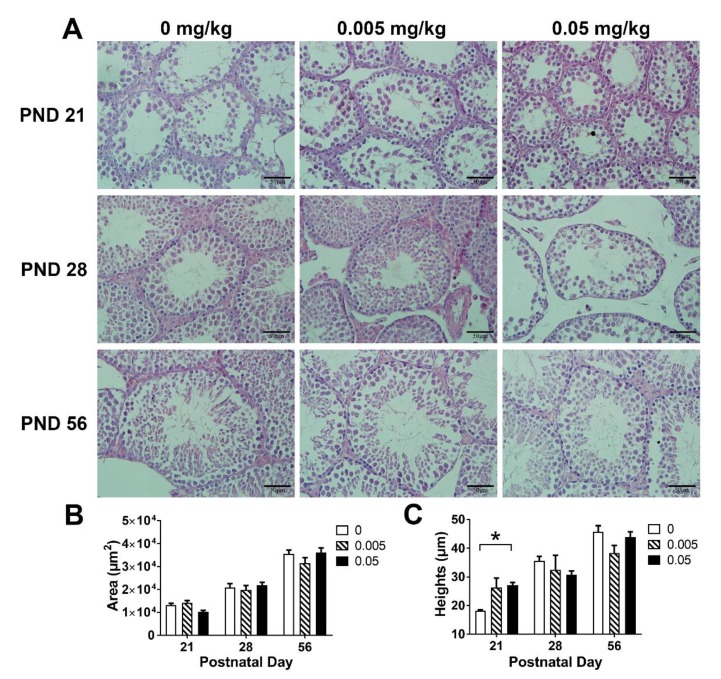
Effects of maternal exposure to T-2 toxin on the testicular histology of offspring. (**A**) Representative images of testicular histology, sections were stained with hematoxylin-eosin (magnification: ×400). (**B**) The cross-sectional area of seminiferous tubules. (**C**) The height of seminiferous epithelium. Each value represents the mean ± SEM of the group (*n* = 3). * *p* < 0.05: compared with the control (0 mg/kg T-2 toxin).

In PND 21, the seminiferous tubule in high T-2 toxin group was smaller as compared with the control, normal seminiferous epithelium damage were detected in both control and low dose of T-2 toxin group; however, there was no significant change in the high dose group (Figure 2A); in PND 28, both low and high dose of T-2 toxin treatment showed significant gaps between the seminiferous tubules; there was no significant difference in the testicular histology among all groups in PND 56. These data indicated that the damage to testicular lesions by T-2 toxin may be alleviated in post-puberty.

The cross-sectional area of seminiferous tubules and the height of seminiferous epithelium were measured to quantify the change in testicular histology (Figure 2B,C). At PND 21, the cross-sectional area of seminiferous tubules in the high dose group tended to decrease as compared with the control (*p* = 0.085), and abnormal seminiferous epithelium increase was detected in the high dose group (*p* < 0.05). There was no significant difference among groups in PND 28 and PND 56. Combined with histomorphological observations, these results indicated that maternal exposure to T-2 toxin may disturb the normal seminiferous tubules and seminiferous epithelium development in both weaning and pre-puberty.

### 3.3. Effects of Maternal Exposure to T-2 Toxin on Antioxidant Indicators in the Testes of Offspring

Maternal exposure to T-2 toxin reduced antioxidant enzyme activity and increased MDA levels in both PND 21 and PND 28 (Table 3). Interestingly, on PND 56, the high dose of T-2 toxin had no significant difference as compared with control, while the low dose of T-2 toxin showed a small increase in antioxidant enzyme activity. A significant dose-dependent effect of 8-OHdG was detected in PND 21, with increased DNA damage at the low dose and no significant changes at the high dose. At PND 56, there was no significant difference among all groups. These results indicated that maternal exposure to T-2 toxin produced significant oxidative stress to the testes of offspring in weaning and pre-puberty, and produced DNA damage in a dose-dependent effect to testes of offspring in weaning. Oxidative stress and DNA damage in T-2 toxin treated groups tended to be normal at post-puberty, it was suggested that the T-2 toxin was cleared by the metabolism of the body itself.

### 3.4. Effects of Maternal Exposure to T-2 Toxin on Testosterone Synthesis in the Testes of Offspring

**Figure 3:**
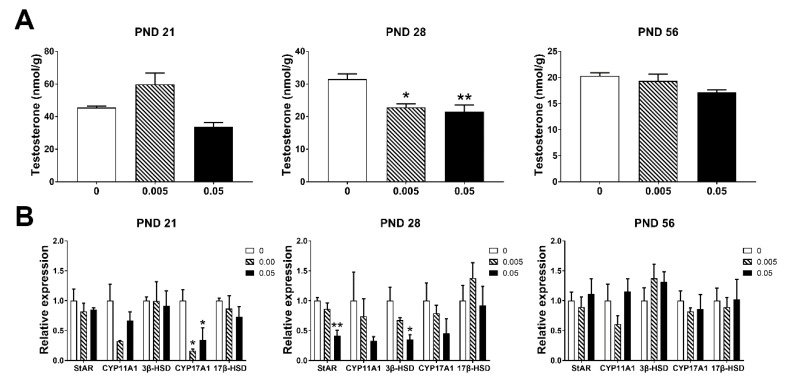
Maternal exposure to T-2 toxin damages the secretion of testosterone and synthesis-related genes in the testes of offspring. (**A**) Testosterone concentration in testes. (**B**) The expression of testosterone synthesis-related genes in testes. Each value represents the mean ± SEM of the group (*n* = 4). * *p* < 0.05, ** *p* < 0.01: compared with the control (0 mg/kg T-2 toxin).

Maternal exposure of T-2 toxin significantly reduced the testosterone concentration in testes at PND 28 (*p* < 0.05), but no significant change in PND 21 and PND 56 (Figure 3A). Maternal exposure to high dose of T-2 toxin down-regulated all testosterone synthesis-related genes in testes of offspring in PND 21 and PND 28; *CYP17A1* was significantly decreased in low dose group in PND 21 (*p* < 0.05); *StAR* and *3β-HSD* were significantly decreased in high dose group as compared to the control in PND 28 (*p* < 0.05). In PND 56, there were no significant changes among all groups (Figure 3B). These results suggested that maternal exposure to T-2 toxin had bad effects on testosterone synthesis in early adolescent mice by down-regulating genes involved in testosterone synthesis. These changes may be associated with high levels of oxidative stress damage detected in PND 21 and PND 28.

### 3.5. Effects of Maternal Exposure of T-2 Toxin on the Expression of Apoptosis-Related Genes in the Testes of Offspring

**Figure 4:**
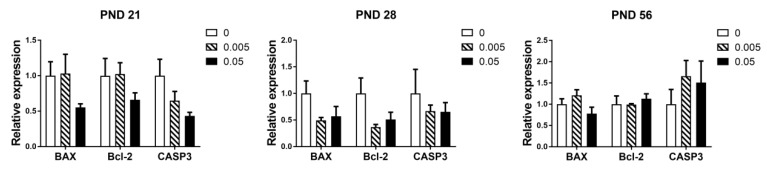
Effects of maternal exposure of T-2 toxin on the expression of apoptosis-related genes in the testes of offspring. Each value represents the mean ± SEM of the group (*n* = 4).

Maternal exposure to T-2 toxin had no significant effects on the expression of genes related to apoptosis in the testes of offspring in all stages (*p* > 0.05). Although we were surprised to find a tendency to decrease the expression of genes related to apoptosis in high dose group at PND 21 and 28, especially the *CASP3* at PND 21 (*p* = 0.054) (Figure 4).

## 4. Discussion

### 4.1. Maternal Exposure to T-2 Toxin Induced Decreases in Body Weight and Testicular Weight

Body weight is a comprehensive indicator for assessing the effects of exposure to toxicants on the general condition of experimental animals. It can reflect the changes in systemic nutritional status and overall function of organisms after sensitive exposure. The decrease in maternal nutritional levels may reduce the birth weight of the offspring, thereby reduce its production performance [21]. In the present study, a significant decrease in body weight and testicular weight was detected in the maternal exposure of T-2 toxin group, and these results were consistent with previous findings of the direct effects of T-2 toxin on mice [16]. The inhibition of the seminiferous tubules’ development (PND 21 histological section) and the increase of the seminiferous tubule gaps (PND 28) may be the cause of testicular weight loss in offspring. These results indicated that maternal exposure to T-2 toxin was toxic to the testicular development of offspring in weaning and pre-puberty; the loss of testicular weight was related to the loss of body weight. Interestingly, there was no significant change in relative testicular weight at weaning day, which indicated that milk of dams may partly protect the testis of offspring.

In post-puberty, when the mice reached sexual maturity, the toxicity of T-2 toxin decreased, indicating that maternal exposure during pregnancy and lactation affects the body weight and organ weight during the initial period of the offspring. After weaning, under sufficient nutrient supply conditions, the compensation growth was achieved, and body weight was restored.

### 4.2. Maternal Exposure to T-2 Toxin Induced Oxidative Stress in the Testes of Offspring During Pre-Puberty

The antioxidant system is an important regulatory system for maintaining spermatogenesis and testosterone synthesis in the testes [19]. During testicular development, the powerful antioxidant system in testis removed large amounts of ROS produced by spermatogenesis and steroidogenesis [30]. The antioxidant capacity of the testis can be reflected by assessing the activity of the relevant enzymes in the antioxidant system. In the testis, the main antioxidant enzymes are SOD, CAT and GPX [31].

T-2 toxin has been shown to decrease the activity of antioxidant enzymes such as SOD, GSH-Px, and CAT mainly via oxidative stress [30]. The latest studies showed that increased oxidative damage was detected in oral T-2 toxin treated mice, with the increase in ROS and MDA and decrease in T-AOC and GSH-Px, accompanied by disordered spermatogenesis [16]. In the present study, maternal exposure to T-2 toxin reduced the activity of SOD and increased the level of MDA in the testes in pre-puberty; moreover, a significant decrease in testicular testosterone was detected in pre-puberty. These results suggested that maternal exposure to T-2 toxin induced significant oxidative damage in the testes of pre-puberty.

8-OHdG is widely accepted as a biomarker for DNA damage induced by oxidative stress [32]. In a normal physiological process, DNA oxidative damage repair mechanism corrects the error of DNA strand through excising 8-OHdG from DNA strand [33]. In previous studies, T-2 toxin induced DNA damage, apoptosis in GH3 cells [34] and articular chondrocyte of rats [35] both via up-regulated expression of 8-OHdG. T-2 toxin induced DNA damage on GH3 cells by increasing the levels of 8-OHdG in a dose-dependent manner. Maternal exposure to T-2 toxin has a dose-dependent effect on 8-OHdG in the testes of weaning mice (PND 21), with a significant increase in low dose group and no effect in high dose group. In contrast, the increase of 8-OhdG in low dose group was abolished at pre-puberty (PND 28), even though a high level of oxidative stress still existed in T-2 toxin group, which indicated that there may be an activated DNA protection mechanism to prevent DNA from oxidative damage in puberty.

### 4.3. Maternal Exposure of T-2 Toxin Inhibited Testosterone Synthesis in the Testes of Offspring During Pre-Puberty

Testosterone has a physiological role in maintaining spermatogenesis, stimulating the development of reproductive organ, maintaining normal libido, and promoting protein synthesis, which is very important in testicular development. In the testis, testosterone is synthesized and secreted by Leydig cells. The biosynthesis of testosterone begins with the transport of cholesterol from the mitochondrial outer membrane to the inner membrane by StAR and then catalyzed by enzymes such as CYP11A1, 3β-HSD, CYP17A1 and 17β-HSD to produce testosterone [36]. In the process of testicular androgen biosynthesis, StAR and CYP11A1 are rate-limiting enzymes, and 3β-HSD and l7β-HSD are key enzymes involved in the formation of sterol hormones.

There are three peak periods of testosterone production by Leydig cells, namely the time between embryonic sex differentiation, birth, and puberty, which induced the differentiation and development of male organs, the hypothalamic masculinization of the central nervous system and the formation of male secondary sexual characteristics respectively [37]. Insufficient testosterone secretion at any period will affect the normal development of the testes. In previous studies, direct exposure of T-2 toxin could inhibit the mRNA expression of 3β-HSD, CYP11A1 and StAR in mouse Leydig cells [38] and impaired testosterone secretion in vitro [39,40] and vivo [17] model. The data detected in the present study showed that after the maternal exposure of T-2 toxin, the genes involved in testosterone synthesis were down-regulated in the testes of pre-puberty offspring, especially StAR, the essential limiting factor in testosterone synthesis. After sexual maturity, there was no significant difference in gene expression involved in testosterone synthesis, and the rapid metabolism of T-2 toxin and the recovery of the body itself may contribute to the recovery. Whether maternal exposure of T-2 toxin has a potential impact on the development of spermatogenesis in testes of offspring remains to be further studied.

### 4.4. Maternal Exposure to T-2 Toxin May Disturb Apoptosis in the Testes of Offspring During Pre-Puberty

The testis is a tissue in which many apoptosis occurs to discard excessive germ cells, or thereby remove germ cells destroyed by toxins [41,42]. Apoptosis is a genetically controlled event, which is regulated by a variety of genes, including the Bcl-2 family, p53, c-Myc genes, and caspases [43,44,45].

BAX is a pro-apoptotic member, while Bcl-2 and Bcl-w belong to the anti-apoptotic member of the Bcl-2 protein family, both of which are involved in early spermatogenesis [46]. CASP3 is considered to be a major executive protease of apoptosis [47]. CASP3 synergistically activates in the first spermatogenic epithelial wave [48], and participates in the regulation of spermatogenic epithelium, sperm differentiation and testicular maturation [49].

In the present study, our results showed that maternal exposure of T-2 toxin has no significant effects on the expression of genes related to apoptosis. Interestingly, in pre-puberty, the offspring of mice maternally exposed to T-2 toxin tended to decrease the expression of apoptosis-related genes. The first time of large-scale apoptosis of testis occurred around 3 weeks after birth, the occurrence of the first apoptotic wave was BAX-related and could be inhibited by the over-expressing of Bcl-2 in the testis of transgenic mice [50]. Combined with the results of histology we could explain the testes’ damage in the control and the low dose, which may indicate the occurrence of the first apoptotic wave on the PND 21. Interestingly, maternal exposure of high dose T-2 toxin had a significant difference as compared with the other two groups in PND 21, these results implied that high dose of T-2 toxin from mother may disturb the first spermatogenic apoptotic wave in the testis, but this hypothesis needs to be further studied.

## 5. Conclusions

The current study indicated that maternal exposure to T-2 toxin had obvious toxic effects on early development at puberty in male offspring. It broke the balance of antioxidant system in testis through oxidative stress, and reduced testosterone synthesis, led to a reduction of testosterone levels at pre-puberty. Although these toxic effects of maternal T-2 exposure on the offspring may be alleviated with the sexual maturity of the offspring, it is worthwhile to investigate the damages caused to offspring at puberty and the potential impacts on spermatogenesis.

## Figures and Tables

**Table 1:** The list of primers for analyzed genes.

Gene	Sequence (5′→3′)	Product Size (bp)
*β-actin* (NM_007393.5)	F: CTGACCGAGCGTGGCTACAG	112
	R: CAGTGGCCATCTCCTGCTCG	
**Testosterone synthesis**		
*StAR* (NM_011485)	F: ACCTCGGTGCTTTAAGGTGA	166
	R: AGCCACAGTGTTTGCTGAAG	
*CYP11A1* (NM_019779.4)	F: TTCTGTGTGGTTAGCGGAGT	91
	R: AGCAAAGTGGATGCAGCAAA	
*3β-HSD* (NM_008293)	F: GATCTGGGCTATGAGCCACT	113
	R: CACTGGCACTTTGTGTCCAA	
*CYP17A1* (NM_007809)	F: CCTTGCTCATCCCACACAAG	127
	R: TCTGGCTGGTCCCATTCATT	
*17β-HSD* (NM_008291)	F: ACCCTTGACCTCCTGAAAGG	146
	R: GGGTTCTTCCAGCATTTCCC	
**Apoptosis**		
*CASP3* (NM_009810)	F: ACGCGCACAAGCTAGAATTT	113
	R: CGGGATCTGTTTCTTTGCGT	
*BAX* (NM_007527)	F: AGGATGATTGCTGACGTGGA	91
	R: CCCAGTTGAAGTTGCCATCA	
*Bcl-2* (NM_009741)	F: TGTCTGGAGCAGGAGAACTG	127
	R: CGGGCTTCTTCTTCTGTGTG	

**Table 2:** Effects of maternal exposure to T-2 toxin on body weight and testicular weight of offspring.

Items	T-2 Toxin (mg/kg Body Weight)		
	0	0.005	0.05
PND 21			
Body weight (g)	11.85 ± 0.64	10.11 ± 0.53	8.52 ± 0.36 **
Testis (mg)	44.96 ± 2.20	37.38 ± 3.75	31.54 ± 2.01 **
Relative testis (%)	0.39 ± 0.03	0.37 ± 0.04	0.37 ± 0.02
PND 28			
Body weight (g)	21.48 ± 0.85	16.59 ± 0.87 **	12.62 ± 0.87 **
Testis (mg)	109.84 ± 3.06	82.62 ± 4.06 **	44.96 ± 2.15 **
Relative testis (%)	0.51 ± 0.01	0.50 ± 0.01	0.36 ± 0.02 **
PND 56			
Body weight (g)	34.28 ± 0.89	33.50 ± 1.19	28.10 ± 1.79 *
Testis (mg)	191.14 ± 5.77	182.88 ±10.68	146.90 ± 5.46 **
Relative testis (%)	0.56 ± 0.01	0.54 ± 0.01	0.53 ± 0.03

Each value represents the mean ± SEM of the group (*n* = 5). * *p* < 0.05, ** *p* < 0.01: compared with the control (0 mg/kg T-2 toxin).

**Table 3:** Effects of maternal exposure to T-2 toxin on the capacity of antioxidant system and DNA damage in the testes of offspring.

Dose	CAT (U/mgprot)	MDA (nmol/mgprot)	SOD (U/mgprot)	GSH-Px (U/mgprot)	8-OHdG (ng/gprot)
(mg/kg)					
PND 21					
0	29.00 ± 3.72	0.45 ± 0.03	395.69 ± 55.09	14.64 ± 3.47	25.24 ± 4.28
0.005	29.04 ± 1.82	0.53 ± 0.01 **	526.81 ± 27.48 **	14.83 ± 4.67	49.94 ± 3.16 **
0.05	14.58 ± 2.82 **	1.22 ± 0.02 **	282.77 ± 14.58 *	4.13 ± 0.52 *	26.03 ± 2.88
PND 28					
0	14.07 ± 1.11	2.06 ± 0.40	329.75 ± 22.31	9.18 ± 2.20	26.04 ± 1.76
0.005	12.60 ± 3.65	2.21 ± 0.24	292.21 ± 15.83	6.66 ± 1.28	20.12 ± 0.81 **
0.05	9.62 ± 1.11	1.86 ± 0.06	215.38 ± 16.05 **	5.76 ± 0.31	15.39 ± 0.48 **
PND 56					
0	23.12 ± 3.65	1.42 ± 0.48	230.43 ± 12.03	6.74 ± 0.48	15.14 ± 0.99
0.005	30.29 ± 4.11	1.88 ± 0.30	263.19 ± 14.59 *	7.80 ± 0.32	17.87 ± 1.39
0.05	22.55 ± 1.22	1.85 ± 0.05	220.58 ± 3.24	6.31 ± 0.60	12.34 ± 1.84

Each value represents the mean ± SEM of the group (*n* = 4). * *p* < 0.05, ** *p* < 0.01: compared with the control (0 mg/kg T-2 toxin).
